# Prophylactic cognitive enhancers for improvement of cognitive function in patients undergoing electroconvulsive therapy: A systematic review and meta-analysis

**DOI:** 10.1097/MD.0000000000019527

**Published:** 2020-03-13

**Authors:** Yunlian Niu, Dan Ye, Yijie You, Jian Wu

**Affiliations:** a Department of Neurology, The First People's Hospital of Changzhou; b Department of Neurology, The Third Affiliated Hospital of Soochow University; c Department of Neurosurgery, The First People's Hospital of Changzhou; d Department of Neurosurgery, The Third Affiliated Hospital of Soochow University, Changzhou, Jiangsu, China.

**Keywords:** cognition, cognitive enhancers, cognitive side effects, ECT, nootropic agents

## Abstract

**Objective:**

Cognitive enhancers, including cholinesterase inhibitors and memantine, are used to treat dementia, but their effect for reducing post-electroconvulsive therapy (post-ECT) cognitive side effects is unclear. We conducted a systematic review and meta-analysis to assess the effectiveness of cognitive enhancers in the prevention of cognitive side effects due to ECT.

**Methods:**

We identified relevant studies by searching electronic databases (e.g., PubMed, EMBASE, Web of Science, Cochrane Library). Only studies published up to October 2019 comparing cognitive enhancer vs placebo for cognitive function after ECT were included. The primary outcome extracted from the studies was cognitive function score.

**Results:**

Five studies with 202 patients were included in this study. The cognitive enhancer group (CEG) had a significantly higher cognitive function score. Moreover, sensitivity analysis showed that no individual study had a significant impact on the overall results.

**Conclusions:**

This meta-analysis revealed that cognitive enhancers might improve cognitive function and reduce ECT-induced cognitive side effects. Nevertheless, more high-quality randomized controlled trials (RCTs) with long-term follow-up are still needed to make the final conclusion.

## Abstract 2

Supplemental Digital Content is available in the text

## 1 Introduction

The use of electroconvulsive therapy (ECT) has historically been demonstrated to be a highly effective and safe treatment method of major depression and catatonic state, especially in patients refractory to pharmacotherapy.^[1,2]^ However, ECT-induced adverse effects have been associated with a variety of transient impairments in cognitive performance and memory, leading to limited use of ECT as an effective treatment of depression, schizophrenia, mania and other conditions where it can potentially produce positive overturning effect.^[3–5]^ The most common adverse effects include anterograde memory impairment, transient delirium and/or retrograde memory impairment.^[6]^ Thus, to improve therapeutic effectiveness, efforts to prevent or limit these cognitive deficits are essential.

One pharmacological approach that has been considered to mitigate adverse effects associated with ECT is the use of cognitive enhancers, agents that are often used to treat dementia.^[5–11]^ These medicines include cholinesterase inhibitor (e.g., donepezil and galantamine) and N-methyl-D-aspartic acid (NMDA) receptor antagonist memantine.^[12]^ Several studies have reported that there is an enhancement of acetylcholinesterase during the postictal period after ECT.^[13,14]^ Therefore, treatment with cholinesterase inhibitors to increase the concentration of acetylcholine, may be useful for the improvement of the cognitive function. In addition, some studies reported the use of NMDA receptor antagonist, including memantine, in the attenuation of ECT-induced cognitive adverse effects.^[9,11]^ To the best of our knowledge, the reason is that memantine modulates the neurotransmitter glutamate.^[15]^

To examine whether cognitive enhancers reduce ECT-related cognitive disorders, we reviewed randomized controlled trials (RCTs) that assessed the efficacy of these cognitive enhancers for prophylaxis against ECT-related cognitive adverse effects compared with placebo therapy in adults undergoing ECT.

## 2 Method

### 2.1 Databases and search strategies

We followed the PRISMA reporting guidelines and the recommendations of Cochrane Collaboration when conducting this meta-analysis. Four electronic databases, namely, PubMed (1966 to October 2019), EMBASE (1980 to October 2019), Cochrane Library (up to October 2019), and Web of Science (1950 to October 2019), were searched for potential studies. The following search key terms were used: cognitive enhancers, cholinesterase inhibitors, cognition disorders, ECT. We used Boolean operators “OR” and “AND” to combine the literature searches (online supplementary appendix 1). The references in full-text articles were manually searched to avoid omitting any potential researches. Literature search was conducted without language restriction. This systematic review and meta-analysis was not registered in the International Prospective Register of Systematic Reviews database.

### 2.2 Inclusion and exclusion criteria

Studies were included in the present meta-analysis if they met the following criteria:

1. patients (aged ≥ 18 years) who need ECT;
2. during the course of ECT, patients received one of four widely available cognitive enhancers (rivastigmine, donepezil, galantamine, memantine), which were compared with placebo;
3. outcomes are measured by the cognitive function score;
4. only RCTs were included.

The exclusion criteria included conference abstracts, case reports, reviews, biochemical trials, retrospective studies, not RCTs, unpublished clinical trials, no assessment of the abovementioned outcomes, or trials without proper treatment groups and control groups.

### 2.3 Study selection and data extraction

Two independent authors (YLN and YJY) followed the unified search strategy to select potentially eligible publications based on articles’ title and abstract. Any conflicts between the reviewers (YLN and YJY) were resolved by discussion or the involvement of a third senior investigator to make a final decision.

The related information in the qualified articles was extracted by two independent reviewers (YLN and YJY) and then cross-checked. A checklist was prepared containing the following items: author name, publication year, source and design of study, patient characteristics, sample size, dosage and type of cognitive enhancers, treatment frequencies and stimulus duration, number of ECT sessions, outcome measurements. On the basis of the results of this research, we chose the following outcomes: cognition function scores were measured by Mini-Mental State Examination (MMSE), Modified Mental Status Examination (3MS) or Repeatable Battery for the Assessment of Neuro-psychological Status (RBANS).^[16–18]^ If incomplete data were encountered, we tried our best to communicate with the corresponding authors for complete data. If the communication failed, we used the guidelines of the Cochrane Handbook for Systematic Reviews of interventions 5.1.0 to extract the data from figures or calculated them.

### 2.4 Quality assessment

The Cochrane risk-of-bias tool was used for evaluating the risk of bias of the included RCTs. The score consisted of seven items, including random sequence generation, allocation concealment, blinding of participants and personnel, blinding of outcome assessment, incomplete outcome data, selective reporting, and other bias.^[19]^ Any inconsistencies between reviewers were resolved through discussion and consensus with a third reviewer.

### 2.5 Statistical analyses

The present study was performed by using Review Manager (Version 5.3.0) software. Standard mean difference (SMD) with the 95% confidence interval (CI) was assessed for continuous outcomes. *P* < .05 was set as the level of significant. Heterogeneity between studies was assessed by using *Q* test and *I*^2^. A fixed-effect model was used when statistically indicated heterogeneity was found (*P* > .1, *I*^2^ < 50%). On the contrary, a random-effect model was used if *P* ≤ .1 or *I*^2^ ≥ 50%, which meant significant heterogeneity. Sensitivity analysis was used to exclude the origins of heterogeneity.

### 2.6 Ethics approval and consent to participate

Ethical approval was not necessary because the present meta-analysis was performed on the basis of previous published studies. Consent for publication was also not necessary because no details, images, or videos relating to individual participants are included in this meta-analysis.

## 3 Result

### 3.1 Study selection

**Figure 1:**
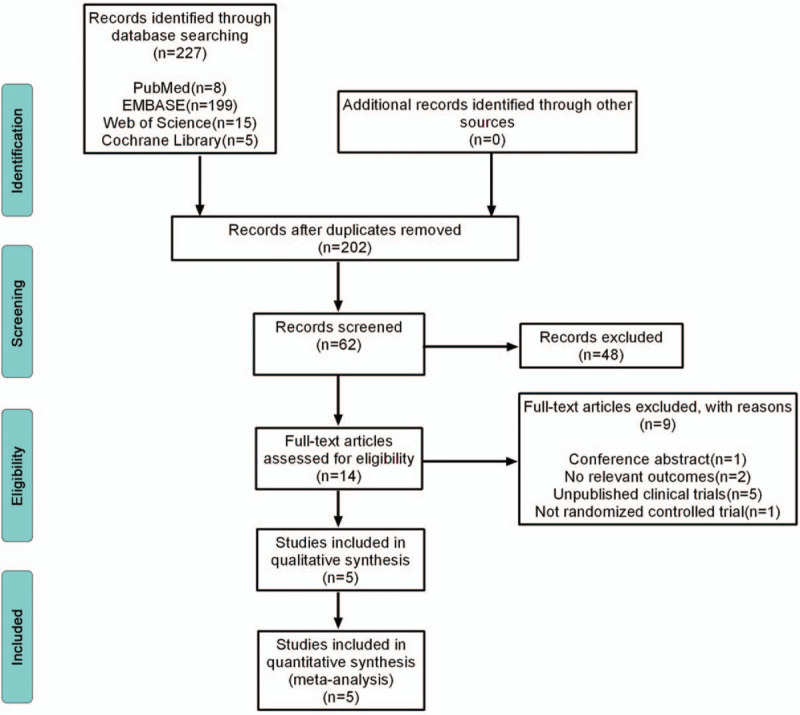
Flow diagram of the study selection process.

By using the key phrases mentioned above, the literature search yielded 227 articles from the following databases: 8 from PubMed, 199 from EMBASE, 15 from Web of Science, and 5 from Cochrane Library. Sixty-two articles were retained after screening and removing duplications, and 48 articles were excluded according to the titles and abstracts. Then, 14 full-text articles were further assessed for eligibility. Of these, 9 articles were excluded on the basis of the exclusion criteria. Finally, 5 RCTs were eligible and included in our systematic review.^[6,10,11,20,21]^ The flow chart of the study selection process is shown in Figure 1.

### 3.2 Study characteristics

The characteristics of the included studies were summarized in Table 1.

**Table 1:**
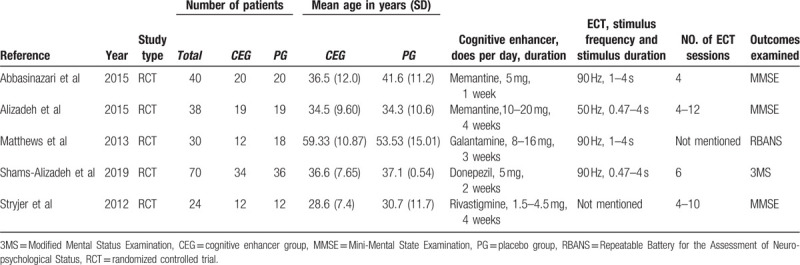
Characteristics of the include studies.

Data were extracted from five RCTs comprising ten treatment arms, which included 202 subjects, with 97 subjects in the cognitive enhancer group (CEG) and 105 subjects in the placebo group (PG). The mean age between the groups was similar, as CEG was 37.99 (28.6–59.33) and 39.54 (30.7–53.53) years in PG. The interventions in the CEG were ECT plus memantine or galantamine or donepezil or rivastigmine. The number of ECT sessions ranged from 4 to 12.^[6,10,11,20,21]^

### 3.3 Risk of bias within studies

**Figure 2:**
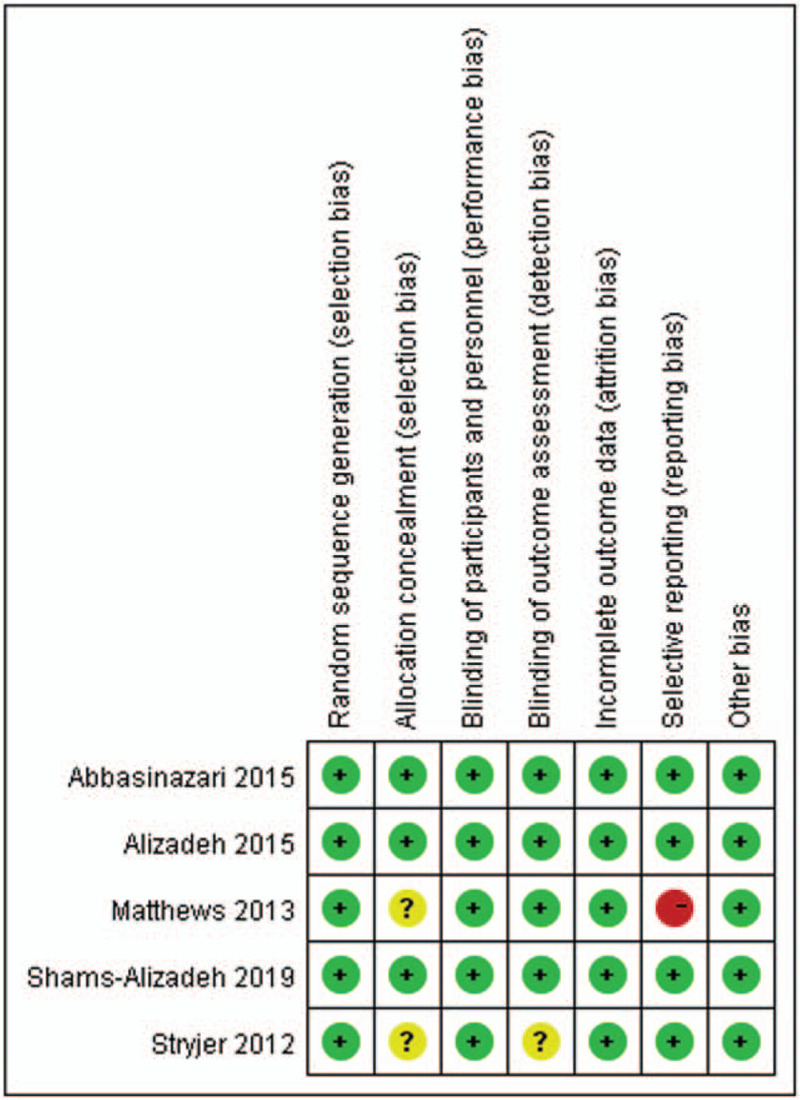
Risk of bias summary.

The risk of bias summary is presented in Figure 2. All of the RCTs described a methodology of randomization. Three studies fully reported the allocation concealment, and the study by Matthews et al did not even describe the allocation concealment. Stryjer et al did not describe the allocation concealment and the blinding of outcome assessment. The blinding and complete outcome data were described in four studies. Those studies were all assessed as having a low overall risk of bias.

### 3.4 Synthesis of results

**Figure 3:**
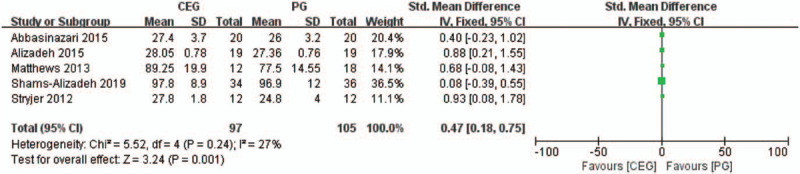
Forest plot comparing cognitive enhancer vs placebo. Outcome: cognitive function changes after treatment.

Five studies provided data that compared cognitive function levels between CEG and PG. Fixed-effect model was utilized to report the pooled effect sizes. The result of the meta-analysis indicated that cognitive function score in CEG was significantly higher than that in PG (SMD = 0.47, 95%CI = 0.18–0.75, *P* = .001, Fig. 3).

### 3.5 Sensitivity analysis

Sensitivity analysis showed that no individual study had a significant impact on the overall results.

## 4 Discussion

Despite ECT has been an effective treatment for psychiatric disorders, ECT-induced cognitive side effects have frequently occurred.^[10]^ To the best of our knowledge, the role of cognitive enhancers in post-ECT cognitive impairment has remained controversial. Currently, some RCTs have suggested that cognitive enhancer as a prophylaxis contributes to protecting memory and improving antidepressant action, as well as relieving cognitive disorder for post-ECT patients.^[10,20,22]^ However, the relevant information on memory and antidepressant action is less well reported and not enough to conduct a systematic review and meta-analysis. Therefore, in the present study we systematically reviewed intervention trials evaluating the usefulness of cognitive enhancers as a pharmacological treatment to prevent or decrease ECT-induced cognitive side effects in its broad definition. Considering the absence of an adequate number of relevant RCTs, further subgroup analyses cannot be performed. Comparing with previous review, this report gives some new superiority:

1. We searched more types of medicine, including cholinesterase inhibitors and NMDA receptor antagonist.
2. We analyzed the effects of cognitive enhancers by quantitative synthesis (meta-analysis).
3. We analyzed more articles to evaluate the impacts of cognitive enhancers for ECT-induced cognitive side effects.^[23]^

By following these principles we aimed at making our systematic review more comprehensive and endowed with a higher confidence level.

Up to present, the majority of the studies showed that patients treated with cognitive enhancers had significantly better post-ECT cognitive function and memory function than patients treated with placebo. Although cognitive enhancers had positive performance, we thought that cognitive enhancers for the reduction of cognitive impairment might not be accurate. First, ECT-induced adverse neurocognitive effects mainly include anterograde amnesia and retrograde amnesia, requiring broad measurement of memory functioning along the course of ECT.^[24,25]^ However, there is no consensus on which diagnostic test would be used for cognitive side effect assessment. Secondly, an adequate study would test retrospective memory function after the acute effects of ECT wore off several weeks after the last ECT. Because the bulk of recovery from ECT cognitive effects occurs during the first 2 weeks, 1 to 2 days is too soon after the ECT course to be clinically useful. For example, Abbasinazari et al found an increase on MMSE, but they did not test retrospective memory and its testing for ECT effects was 1 day after the fourth ECT.^[20]^ Alizadeh et al reported that cognitive performance was enhanced in patients receiving memantine during ECT. However, they only tested immediate memory and its post-ECT testing was 24 h after ECT.^[11]^ With a large number of testing, Matthews et al studied anterograde memory function only 1 to 2 days after the final ECT. Their study reported that only delayed memory showed an effect associated with galantamine administration accompanying ECT.^[6]^ Shams-Alizadeh et al reported that donepezil had no effect on cognitive deficits. However, their post-ECT testing was done only 48 h after the last ECT.^[10]^ Only Stryjer et al assessed long-range cognitive functioning and revealed negative results 4 weeks after the last ECT session.^[21]^ Thirdly, it was noteworthy that most of their papers focused on the MMSE score. Although one MMSE item examines immediate recall, this is a very incomplete examination of memory function. Because of the issue, we could not further analyze the effect of cognitive enhancers on different aspects of memory function. Moreover, a new study reported that MMSE score increases along a course of ECT, and this showed the disconnect between MMSE score and appropriate concern about adverse cognitive effects of ECT.^[26]^ To solve those issues, in this systematic review, all types of studies are RCTs, which creates a similar experimental condition between the 2 groups. In contrast, we used the total MMSE score for analysis in 48 h after the final ECT. Although the major issue evaluated in those studies included an increase of MMSE score during ECT and short follow-up period, at least the majority of studies in this review demonstrated a significantly higher total MMSE score in CEG. Therefore, we have reasons to believe that our meta-analysis appropriately decreases the disconnect between MMSE score and cognitive side effects of ECT. Taken together, we tried to complete a meta-analysis based on all reasons. We found that cognitive function score in CEG was significantly higher than that in PG. Because statistical significant does not equal clinical relevance, we suggested that a larger battery of cognitive measures should be used to acquire full insight in cognitive functioning, underlining the need to further verify the beneficial effects of cognitive enhancers to decrease ECT-induced side effects.

## 5 Limitations

The systematic review and meta-analysis reported here combines data across studies in order to evaluate therapeutic effects with more reliability than is possible in a single. Actually, our review has several limitations. First, the main limitation of this study, as with other reviews, is that the subject population, the cognitive enhancer's kind and the outcome definitions are not the same across studies. Secondly, variations in electrode placement and stimulus dosing have large effects on cognitive changes from ECT, particularly on retrograde memory, but they are not mentioned in those articles, a notable omission. Thirdly, different age determines different recovery rates of cognition, so we believe that age plays a significant role in reducing ECT-induced cognitive side effects.^[27]^ Unfortunately, this point was ignored in most of the studies.^[11,20,21]^ It follows that the results might not be applicable to patients of all ages. Fourthly, RCTs did not evaluate enough patients or did not follow patients for a sufficient duration to allow a definitive conclusion that make generalization difficult. Fifthly, the assessment of the clinical relevance of the results of the reviewed studies is still challengeable due to the imperfection of post-ECT cognitive assessment. Finally, when beginning this review, we planned to find a mass of RCTs that would allow us to determine the effectiveness and safety of cognitive enhancers for ECT-induced cognitive impairment. However, our extensive literature searches only identified 5 studies. Our study may not be considered broadly representative because of the small number of relevant studies included in the analysis. Although all results point into the same direction: Cognitive enhancers provide a significantly better post-ECT cognitive performance during ECT. The results may seem limited due to the imperfections in the studies. In summary, due to limited evidence, we emphasize the need to further explore the potential treatment benefits of cognitive enhancers for ECT-induced cognitive side effects.

## 6 Conclusions

The present systematic review and meta-analysis assessed the preventive effect of cognitive enhancers on ECT-induced cognitive deficits and provided a thorough synthesis of results from RCTs. The systematic review and meta-analysis indicated that the addition of cognitive enhancers might have clinical effectiveness for improving the score of cognitive function in patients undergoing ECT. Even though cognitive enhancers appear promising for this indication as a category, among the 5 included trials only 2 papers reported a statistically significant effect as compared with placebo for the specified endpoint: one paper investigating memantine and one investigating rivastigmine, respectively. The ones investigating donepezil, galantamine and one on memantine failed to demonstrate a significant difference from placebo. However, considering the overall results yielded by our analysis and the previously mentioned limitations of the included studies, we conclude that these results should be regarded as promising, emphasizing the need to further explore the potential treatment benefits of cognitive enhancers for ECT-induced cognitive side effects in order to expand the evidence supporting their usefulness.

## Acknowledgments

We would like to give sincere appreciation to the reviewers for their many useful comments on the early version of the manuscript.

## Author contributions

**Conceptualization:** Yijie You.

**Data curation:** Yunlian Niu, Yijie You, Dan Ye.

**Formal analysis:** Yunlian Niu, Yijie You.

**Investigation:** Yunlian Niu, Yijie You.

**Methodology:** Yunlian Niu, Yijie You.

**Project administration:** Yijie You, Jian Wu.

**Software:** Yunlian Niu, Yijie You.

**Supervision:** Yijie You.

**Validation:** Yijie You, Jian Wu.

**Writing – original draft:** Yunlian Niu.

**Writing – review and editing:** Yunlian Niu, Yijie You.

Yijie You orcid: 0000-0003-4062-4347.

## Supplementary Material

Supplemental Digital Content
