# Supplementary material for: Prophylactic cognitive enhancers for improvement of cognitive function in patients undergoing electroconvulsive therapy: A systematic review and meta-analysis
Source: Medicine (Baltimore). 2020 Mar 13;99(11):e19527. doi: 10.1097/MD.0000000000019527 (PMC7440112; doi:10.1097/MD.0000000000019527)
Supplement: Supplemental Digital Content [file medi-99-e19527-s001.pdf]

Appendix 1: Search terms and strategies

**PubMed search strategy**	
#1	"Nootropic Agents"[Mesh]
#2	("Nootropic Agents"[Mesh]) OR (Nootropic Agents[Title/Abstract] or Agents, Nootropic[Title/Abstract] or Nootropic Drugs[Title/Abstract] or Drugs, Nootropic[Title/Abstract] or Procognitive Agents[Title/Abstract] or Agents, Procognitive[Title/Abstract] or Nootropics[Title/Abstract] or Anti-Dementia Agents[Title/Abstract] or Agents, Anti-Dementia[Title/Abstract] or Anti Dementia Agents[Title/Abstract] or Antidementia Agents[Title/Abstract] or Agents, Antidementia[Title/Abstract] or Cognitive Enhancers[Title/Abstract] or Enhancers, Cognitive[Title/Abstract])
#3	"Cholinesterase Inhibitors"[Mesh]
#4	("Cholinesterase Inhibitors"[Mesh]) OR (Cholinesterase Inhibitors[Title/Abstract] or Inhibitors, Cholinesterase[Title/Abstract] or Anticholinesterase Drugs[Title/Abstract] or Drugs, Anticholinesterase[Title/Abstract] or Anticholinesterases[Title/Abstract] or Anticholinesterase Agents[Title/Abstract] or Agents, Anticholinesterase[Title/Abstract] or Anti-Cholinesterases[Title/Abstract] or Anti Cholinesterases[Title/Abstract] or Cholinesterase Inhibitors, Irreversible[Title/Abstract] or Inhibitors, Irreversible Cholinesterase[Title/Abstract] or Irreversible Cholinesterase Inhibitors[Title/Abstract] or Cholinesterase Inhibitors, Reversible[Title/Abstract] or Inhibitors, Reversible Cholinesterase[Title/Abstract] or Reversible Cholinesterase Inhibitors[Title/Abstract] or Acetylcholinesterase Inhibitors[Title/Abstract] or Inhibitors, Acetylcholinesterase[Title/Abstract])
#5	"Electroconvulsive Therapy"[Mesh]
#6	("Electroconvulsive Therapy"[Mesh]) OR (Electroconvulsive Therapy[Title/Abstract] or Electroconvulsive Therapies[Title/Abstract] or Therapies, Electroconvulsive[Title/Abstract] or Therapy, Electroconvulsive[Title/Abstract] or Electroshock Therapy[Title/Abstract] or Electroshock Therapies[Title/Abstract] or Therapies, Electroshock[Title/Abstract] or Therapy, Electroshock[Title/Abstract] or Convulsive Therapy, Electric[Title/Abstract] or Convulsive Therapies, Electric[Title/Abstract] or Electric Convulsive Therapies[Title/Abstract] or Electric Convulsive Therapy[Title/Abstract] or Therapies, Electric Convulsive[Title/Abstract] or Therapy, Electric Convulsive[Title/Abstract] or Shock Therapy, Electric[Title/Abstract] or Electric Shock Therapies[Title/Abstract] or Electric Shock Therapy[Title/Abstract] or Shock Therapies, Electric[Title/Abstract] or Therapies, Electric Shock[Title/Abstract] or Therapy, Electric Shock[Title/Abstract] or ECT (Psychotherapy)[Title/Abstract])
#7	((("Cholinesterase Inhibitors"[Mesh]) OR (Cholinesterase Inhibitors[Title/Abstract] or Inhibitors, Cholinesterase[Title/Abstract] or Anticholinesterase Drugs[Title/Abstract] or Drugs, Anticholinesterase[Title/Abstract] or Anticholinesterases[Title/Abstract] or Anticholinesterase Agents[Title/Abstract] or Agents, Anticholinesterase[Title/Abstract] or Anti-Cholinesterases[Title/Abstract] or Anti Cholinesterases[Title/Abstract] or Cholinesterase Inhibitors, Irreversible[Title/Abstract] or Inhibitors, Irreversible Cholinesterase[Title/Abstract] or Irreversible Cholinesterase Inhibitors[Title/Abstract] or Cholinesterase Inhibitors, Reversible[Title/Abstract] or Inhibitors, Reversible Cholinesterase[Title/Abstract] or Reversible Cholinesterase Inhibitors[Title/Abstract] or Acetylcholinesterase Inhibitors[Title/Abstract] or Inhibitors, Acetylcholinesterase[Title/Abstract]))) OR (("Nootropic Agents"[Mesh]) OR (Nootropic Agents[Title/Abstract] or Agents, Nootropic[Title/Abstract] or Nootropic Drugs[Title/Abstract] or Drugs, Nootropic[Title/Abstract] or Procognitive Agents[Title/Abstract] or Agents, Procognitive[Title/Abstract] or Nootropics[Title/Abstract] or Anti-Dementia Agents[Title/Abstract] or Agents, Anti-Dementia[Title/Abstract] or Anti Dementia Agents[Title/Abstract] or Antidementia Agents[Title/Abstract] or Agents, Antidementia[Title/Abstract] or Cognitive Enhancers[Title/Abstract] or Enhancers, Cognitive[Title/Abstract]))
#8	"Cognitive Dysfunction"[Mesh]
#9	("Cognitive Dysfunction"[Mesh]) OR (Cognitive Dysfunction[Title/Abstract] or Cognitive Dysfunctions[Title/Abstract] or Dysfunction, Cognitive[Title/Abstract] or Dysfunctions, Cognitive[Title/Abstract] or Cognitive Impairments[Title/Abstract] or Cognitive Impairment[Title/Abstract] or Impairment, Cognitive[Title/Abstract] or Impairments, CognitiveMild[Title/Abstract] or Cognitive Impairment[Title/Abstract] or Cognitive Impairment, Mild[Title/Abstract] or Cognitive Impairments, Mild[Title/Abstract] or Impairment, Mild Cognitive[Title/Abstract] or Impairments, Mild Cognitive[Title/Abstract] or Mild Cognitive Impairments[Title/Abstract] or Mild Neurocognitive Disorder[Title/Abstract] or Disorder, Mild Neurocognitive[Title/Abstract] or Disorders, Mild Neurocognitive[Title/Abstract] or Mild Neurocognitive Disorders[Title/Abstract] or Neurocognitive Disorder, Mild[Title/Abstract] or Neurocognitive Disorders, Mild[Title/Abstract] or Cognitive Decline[Title/Abstract] or Cognitive Declines[Title/Abstract] or Decline, Cognitive[Title/Abstract] or Declines, Cognitive[Title/Abstract] or Mental Deterioration[Title/Abstract] or Deterioration, Mental[Title/Abstract] or Deteriorations, Mental[Title/Abstract] or Mental Deteriorations[Title/Abstract])
#10	((((((("Cholinesterase Inhibitors"[Mesh]) OR (Cholinesterase Inhibitors[Title/Abstract] or Inhibitors, Cholinesterase[Title/Abstract] or Anticholinesterase Drugs[Title/Abstract] or Drugs, Anticholinesterase[Title/Abstract] or Anticholinesterases[Title/Abstract] or Anticholinesterase Agents[Title/Abstract] or Agents, Anticholinesterase[Title/Abstract] or Anti-Cholinesterases[Title/Abstract] or Anti Cholinesterases[Title/Abstract] or Cholinesterase Inhibitors, Irreversible[Title/Abstract] or Inhibitors, Irreversible Cholinesterase[Title/Abstract] or Irreversible Cholinesterase Inhibitors[Title/Abstract] or Cholinesterase Inhibitors, Reversible[Title/Abstract] or Inhibitors, Reversible Cholinesterase[Title/Abstract] or Reversible Cholinesterase Inhibitors[Title/Abstract] or Acetylcholinesterase Inhibitors[Title/Abstract] or Inhibitors, Acetylcholinesterase[Title/Abstract]))) OR (("Nootropic Agents"[Mesh]) OR (Nootropic Agents[Title/Abstract] or Agents, Nootropic[Title/Abstract] or Nootropic Drugs[Title/Abstract] or Drugs, Nootropic[Title/Abstract] or Procognitive Agents[Title/Abstract] or Agents, Procognitive[Title/Abstract] or Nootropics[Title/Abstract] or Anti-Dementia Agents[Title/Abstract] or Agents, Anti-Dementia[Title/Abstract] or Anti Dementia Agents[Title/Abstract] or Antidementia Agents[Title/Abstract] or Agents, Antidementia[Title/Abstract] or Cognitive Enhancers[Title/Abstract] or Enhancers, Cognitive[Title/Abstract])))) AND (("Electroconvulsive Therapy"[Mesh]) OR (Electroconvulsive Therapy[Title/Abstract] or Electroconvulsive Therapies[Title/Abstract] or Therapies, Electroconvulsive[Title/Abstract] or Therapy, Electroconvulsive[Title/Abstract] or Electroshock Therapy[Title/Abstract] or Electroshock Therapies[Title/Abstract] or Therapies, Electroshock[Title/Abstract] or Therapy, Electroshock[Title/Abstract] or Convulsive Therapy, Electric[Title/Abstract] or Convulsive Therapies, Electric[Title/Abstract] or Electric Convulsive Therapies[Title/Abstract] or Electric Convulsive Therapy[Title/Abstract] or Therapies, Electric Convulsive[Title/Abstract] or Therapy, Electric Convulsive[Title/Abstract] or Shock Therapy, Electric[Title/Abstract] or Electric Shock Therapies[Title/Abstract] or Electric Shock Therapy[Title/Abstract] or Shock Therapies, Electric[Title/Abstract] or Therapies, Electric Shock[Title/Abstract] or Therapy, Electric Shock[Title/Abstract] or ECT (Psychotherapy)[Title/Abstract])))) AND (("Cognitive Dysfunction"[Mesh]) OR (Cognitive Dysfunction[Title/Abstract] or Cognitive Dysfunctions[Title/Abstract] or Dysfunction, Cognitive[Title/Abstract] or Dysfunctions, Cognitive[Title/Abstract] or Cognitive Impairments[Title/Abstract] or Cognitive Impairment[Title/Abstract] or Impairment, Cognitive[Title/Abstract] or Impairments, CognitiveMild[Title/Abstract] or Cognitive Impairment[Title/Abstract] or Cognitive Impairment, Mild[Title/Abstract] or Cognitive Impairments, Mild[Title/Abstract] or Impairment, Mild Cognitive[Title/Abstract] or Impairments, Mild Cognitive[Title/Abstract] or Mild Cognitive Impairments[Title/Abstract] or Mild Neurocognitive Disorder[Title/Abstract] or Disorder, Mild Neurocognitive[Title/Abstract] or Disorders, Mild Neurocognitive[Title/Abstract] or Mild Neurocognitive Disorders[Title/Abstract] or Neurocognitive Disorder, Mild[Title/Abstract] or Neurocognitive Disorders, Mild[Title/Abstract] or Cognitive Decline[Title/Abstract] or Cognitive Declines[Title/Abstract] or Decline, Cognitive[Title/Abstract] or Declines, Cognitive[Title/Abstract] or Mental Deterioration[Title/Abstract] or Deterioration, Mental[Title/Abstract] or Deteriorations, Mental[Title/Abstract] or Mental Deteriorations[Title/Abstract]))
**EMBASE search strategy**	
#1	'nootropic agent'/exp
#2	'nootropic agents':ab,ti OR 'agents, nootropic':ab,ti OR 'nootropic drugs':ab,ti OR 'drugs, nootropic':ab,ti OR 'procognitive agents':ab,ti OR 'agents, procognitive':ab,ti OR 'nootropics':ab,ti OR 'anti-dementia agents':ab,ti OR 'agents, anti-dementia':ab,ti OR 'anti dementia agents':ab,ti OR 'antidementia agents':ab,ti OR 'agents, antidementia':ab,ti OR 'cognitive enhancers':ab,ti OR 'enhancers, cognitive':ab,ti
#3	#1 OR #2
#4	'cholinesterase inhibitor'/exp
#5	'cholinesterase inhibitors':ab,ti OR 'inhibitors, cholinesterase':ab,ti OR 'anticholinesterase drugs':ab,ti OR 'drugs, anticholinesterase':ab,ti OR 'anticholinesterases':ab,ti OR 'anticholinesterase agents':ab,ti OR 'agents, anticholinesterase':ab,ti OR 'anti-cholinesterases':ab,ti OR 'anti cholinesterases':ab,ti OR 'cholinesterase inhibitors, irreversible':ab,ti OR 'inhibitors, irreversible cholinesterase':ab,ti OR 'irreversible cholinesterase inhibitors':ab,ti OR 'cholinesterase inhibitors, reversible':ab,ti OR 'inhibitors, reversible cholinesterase':ab,ti OR 'reversible cholinesterase inhibitors':ab,ti OR 'acetylcholinesterase inhibitors':ab,ti OR 'inhibitors, acetylcholinesterase':ab,ti
#6	#4 OR #5
#7	'electroconvulsive therapy'/exp
#8	'electroconvulsive therapy':ab,ti OR 'electroconvulsive therapies':ab,ti OR 'therapies, electroconvulsive':ab,ti OR 'therapy, electroconvulsive':ab,ti OR 'electroshock therapy':ab,ti OR 'electroshock therapies':ab,ti OR 'therapies, electroshock':ab,ti OR 'therapy, electroshock':ab,ti OR 'convulsive therapy, electric':ab,ti OR 'convulsive therapies, electric':ab,ti OR 'electric convulsive therapies':ab,ti OR 'electric convulsive therapy':ab,ti OR 'therapies, electric convulsive':ab,ti OR 'therapy, electric convulsive':ab,ti OR 'shock therapy, electric':ab,ti OR 'electric shock therapies':ab,ti OR 'electric shock therapy':ab,ti OR 'shock therapies, electric':ab,ti OR 'therapies, electric shock':ab,ti OR 'therapy, electric shock':ab,ti OR 'ect (psychotherapy)':ab,ti
#9	#7 OR #8
#10	cognitive defect'/exp
#11	cognitive dysfunction':ab,ti OR 'cognitive dysfunctions':ab,ti OR 'dysfunction, cognitive':ab,ti OR 'dysfunctions, cognitive':ab,ti OR 'cognitive impairments':ab,ti OR 'impairment, cognitive':ab,ti OR 'impairments, cognitivemild':ab,ti OR 'cognitive impairment':ab,ti OR 'cognitive impairment, mild':ab,ti OR 'cognitive impairments, mild':ab,ti OR 'impairment, mild cognitive':ab,ti OR 'impairments, mild cognitive':ab,ti OR 'mild cognitive impairments':ab,ti OR 'mild neurocognitive disorder':ab,ti OR 'disorder, mild neurocognitive':ab,ti OR 'disorders, mild neurocognitive':ab,ti OR 'mild neurocognitive disorders':ab,ti OR 'neurocognitive disorder, mild':ab,ti OR 'neurocognitive disorders, mild':ab,ti OR 'cognitive decline':ab,ti OR 'cognitive declines':ab,ti OR 'decline, cognitive':ab,ti OR 'declines, cognitive':ab,ti OR 'mental deterioration':ab,ti OR 'deteriorations, mental':ab,ti OR 'mental deteriorations':ab,ti
#12	#10 OR #11
#13	#2 OR #6
#14	#9 AND #12 AND #13
**Web of Science search strategy**	
#1	TS=(Nootropic Agents or Nootropic Agents* or Agents, Nootropic* or Nootropic Drugs* or Drugs, Nootropic* or Procognitive Agents* or Agents, Procognitive* or Nootropics* or Anti-Dementia Agents* or Agents, Anti-Dementia* or Anti Dementia Agents* or Antidementia Agents* or Agents, Antidementia* or Cognitive Enhancers* or Enhancers, Cognitive*)
#2	TS=(Cholinesterase Inhibitors or Cholinesterase Inhibitors* or Inhibitors, Cholinesterase* or Anticholinesterase Drugs* or Drugs, Anticholinesterase* or Anticholinesterases* or Anticholinesterase Agents* or Agents, Anticholinesterase* or Anti-Cholinesterases* or Anti Cholinesterases* or Cholinesterase Inhibitors, Irreversible* or Inhibitors, Irreversible Cholinesterase* or Irreversible Cholinesterase Inhibitors* or Cholinesterase Inhibitors, Reversible* or Inhibitors, Reversible Cholinesterase* or Reversible Cholinesterase Inhibitors* or Acetylcholinesterase Inhibitors* or Inhibitors, Acetylcholinesterase*)
#3	TS=(Electroconvulsive Therapy or Electroconvulsive Therapy* or Electroconvulsive Therapies* or Therapies, Electroconvulsive* or Therapy, Electroconvulsive* or Electroshock Therapy* or Electroshock Therapies* or Therapies, Electroshock* or Therapy, Electroshock* or Convulsive Therapy, Electric* or Convulsive Therapies, Electric* or Electric Convulsive Therapies* or Electric Convulsive Therapy* or Therapies, Electric Convulsive* or Therapy, Electric Convulsive* or Shock Therapy, Electric* or Electric Shock Therapies* or Electric Shock Therapy* or Shock Therapies, Electric* or Therapies, Electric Shock* or Therapy, Electric Shock* or ECT (Psychotherapy))
#4	TS=(Cognitive Dysfunction or Cognitive Dysfunction* or Cognitive Dysfunctions* or Dysfunction, Cognitive* or Dysfunctions, Cognitive* or Cognitive Impairments* or Cognitive Impairment* or Impairment, Cognitive* or Impairments, CognitiveMild* or Cognitive Impairment* or Cognitive Impairment, Mild* or Cognitive Impairments, Mild* or Impairment, Mild Cognitive* or Impairments, Mild Cognitive* or Mild Cognitive Impairments* or Mild Neurocognitive Disorder* or Disorder, Mild Neurocognitive* or Disorders, Mild Neurocognitive* or Mild Neurocognitive Disorders* or Neurocognitive Disorder, Mild* or Neurocognitive Disorders, Mild* or Cognitive Decline* or Cognitive Declines* or Decline, Cognitive* or Declines, Cognitive* or Mental Deterioration* or Deterioration, Mental* or Deteriorations, Mental* or Mental Deteriorations*)
#5	#2 OR #1
#6	#5 AND #4 AND #3
**Cochrane Library search strategy**	
#1	MeSH descriptor: [Nootropic Agents] explode all trees
#2	(Nootropic Agents):ti,ab,kw OR (Agents, Nootropic):ti,ab,kw OR (Nootropic Drugs):ti,ab,kw OR (Drugs, Nootropic):ti,ab,kw OR (Procognitive Agents):ti,ab,kw OR (Agents, Procognitive):ti,ab,kw OR (Nootropics):ti,ab,kw OR (Anti-Dementia Agents):ti,ab,kw OR (Agents, Anti-Dementia):ti,ab,kw OR (Anti Dementia Agents):ti,ab,kw OR (Antidementia Agents):ti,ab,kw OR (Agents, Antidementia):ti,ab,kw OR (Cognitive Enhancers):ti,ab,kw OR (Enhancers, Cognitive):ti,ab,kw
#3	#1 or #2
#4	MeSH descriptor: [Cholinesterase Inhibitors] explode all trees
#5	(Cholinesterase Inhibitors):ti,ab,kw OR (Inhibitors, Cholinesterase):ti,ab,kw OR (Anticholinesterase Drugs):ti,ab,kw OR (Drugs, Anticholinesterase):ti,ab,kw OR (Anticholinesterases):ti,ab,kw OR (Anticholinesterase Agents):ti,ab,kw OR (Agents, Anticholinesterase):ti,ab,kw OR (Anti-Cholinesterases):ti,ab,kw OR (Anti Cholinesterases):ti,ab,kw OR (Cholinesterase Inhibitors, Irreversible):ti,ab,kw OR (Inhibitors, Irreversible Cholinesterase):ti,ab,kw OR (Irreversible Cholinesterase Inhibitors):ti,ab,kw OR (Cholinesterase Inhibitors, Reversible):ti,ab,kw OR (Inhibitors, Reversible Cholinesterase):ti,ab,kw OR (Reversible Cholinesterase Inhibitors):ti,ab,kw OR (Acetylcholinesterase Inhibitors):ti,ab,kw OR (Inhibitors, Acetylcholinesterase):ti,ab,kw
#6	#4 or #5
#7	MeSH descriptor: [Electroconvulsive Therapy] explode all trees
#8	(Electroconvulsive Therapy):ti,ab,kw OR (Electroconvulsive Therapies):ti,ab,kw OR (Therapies, Electroconvulsive):ti,ab,kw OR (Therapy, Electroconvulsive):ti,ab,kw OR (Electroshock Therapy):ti,ab,kw OR (Electroshock Therapies):ti,ab,kw OR (Therapies, Electroshock):ti,ab,kw OR (Therapy, Electroshock):ti,ab,kw OR (Convulsive Therapy, Electric):ti,ab,kw OR (Convulsive Therapies, Electric):ti,ab,kw OR (Electric Convulsive Therapies):ti,ab,kw OR (Electric Convulsive Therapy):ti,ab,kw OR (Therapies, Electric Convulsive):ti,ab,kw OR (Therapy, Electric Convulsive):ti,ab,kw OR (Shock Therapy, Electric):ti,ab,kw OR (Electric Shock Therapies):ti,ab,kw OR (Electric Shock Therapy):ti,ab,kw OR (Shock Therapies, Electric):ti,ab,kw OR (Therapies, Electric Shock):ti,ab,kw OR (Therapy, Electric Shock):ti,ab,kw OR (ECT (Psychotherapy)):ti,ab,kw
#9	#7 or #8
#10	MeSH descriptor: [Cognitive Dysfunction] explode all trees
#11	(Cognitive Dysfunction):ti,ab,kw OR (Cognitive Dysfunctions):ti,ab,kw OR (Dysfunction, Cognitive):ti,ab,kw OR (Dysfunctions, Cognitive):ti,ab,kw OR (Cognitive Impairments):ti,ab,kw OR (Cognitive Impairment):ti,ab,kw OR (Impairment, Cognitive):ti,ab,kw OR (Impairments, CognitiveMild):ti,ab,kw OR (Cognitive Impairment):ti,ab,kw OR (Cognitive Impairment, Mild):ti,ab,kw OR (Cognitive Impairments, Mild):ti,ab,kw OR (Impairment, Mild Cognitive):ti,ab,kw OR (Impairments, Mild Cognitive):ti,ab,kw OR (Mild Cognitive Impairments):ti,ab,kw OR (Mild Neurocognitive Disorder):ti,ab,kw OR (Disorder, Mild Neurocognitive):ti,ab,kw OR (Disorders, Mild Neurocognitive):ti,ab,kw OR (Mild Neurocognitive Disorders):ti,ab,kw OR (Neurocognitive Disorder, Mild):ti,ab,kw OR (Neurocognitive Disorders, Mild):ti,ab,kw OR (Cognitive Decline):ti,ab,kw OR (Cognitive Declines):ti,ab,kw OR (Decline, Cognitive):ti,ab,kw OR (Declines, Cognitive):ti,ab,kw OR (Mental Deterioration):ti,ab,kw OR (Deterioration, Mental):ti,ab,kw OR (Deteriorations, Mental):ti,ab,kw OR (Mental Deteriorations):ti,ab,kw
#12	#10 or #11
#13	#3 or #6
#14	#9 and #12 and #13
